# (2,2′-Bipyridine-κ^2^ *N*,*N*′)dibromido(dimethyl sulfoxide-κ*O*)zinc(II)

**DOI:** 10.1107/S1600536810017551

**Published:** 2010-05-22

**Authors:** Majid Esmhosseini

**Affiliations:** a Department of Chemistry, University of Urmiyeh, Urmyieh, Iran

## Abstract

In the mol­ecule of the title compound, [ZnBr_2_(C_10_H_8_N_2_)(C_2_H_6_OS)], the Zn^II^ atom is five-coordinated in a distorted trigonal–bipyramidal configuration by two N atoms from one 2,2′-bipyridine, one O atom from one dimethyl­sulfoxide molecule and two Br atoms. Inter­molecular π–π stacking between parallel pyridine rings [face-to-face distance 3.32 (4) Å] and C—H⋯Br and C—H⋯O hydrogen-bonding interactions are present in the crystal structure.

## Related literature

For related structures, see: Ahmadi *et al.* (2008 ▶); Alizadeh, Kalateh, Ebadi *et al.* (2009 ▶); Alizadeh, Kalateh, Khoshtarkib *et al.* (2009 ▶); Alizadeh, Khoshtarkib *et al.* (2009 ▶); Blake *et al.* (2007 ▶); Khan & Tuck (1984 ▶); Marjani *et al.* (2007 ▶); Khalighi *et al.* (2008 ▶).
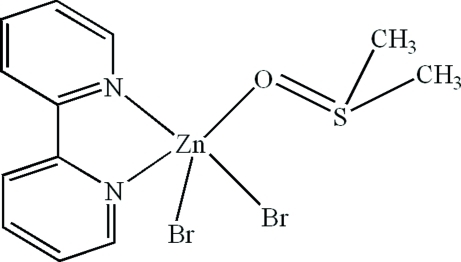


## Experimental

### 

#### Crystal data

[ZnBr_2_(C_10_H_8_N_2_)(C_2_H_6_OS)]
*M* *_r_* = 459.51
Monoclinic, 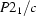

*a* = 9.4802 (10) Å
*b* = 8.3449 (7) Å
*c* = 19.989 (2) Å
β = 95.185 (8)°
*V* = 1574.9 (3) Å^3^
*Z* = 4
Mo *K*α radiation
μ = 6.76 mm^−1^
*T* = 298 K
0.30 × 0.25 × 0.20 mm

#### Data collection

Bruker SMART CCD area-detector diffractometer
Absorption correction: multi-scan (*SADABS*; Bruker, 2001 ▶) *T*_min_ = 0.148, *T*_max_ = 0.260
12674 measured reflections
4245 independent reflections
3229 reflections with *I* > 2σ(*I*)
*R*_int_ = 0.078

#### Refinement

*R*[*F* ^2^ > 2σ(*F* ^2^)] = 0.058
*wR*(*F* ^2^) = 0.140
*S* = 1.13
4245 reflections
175 parameters
H-atom parameters constrained
Δρ_max_ = 1.08 e Å^−3^
Δρ_min_ = −1.46 e Å^−3^

### 

Data collection: *SMART* (Bruker, 1998 ▶); cell refinement: *SAINT* (Bruker, 1998 ▶); data reduction: *SAINT*; program(s) used to solve structure: *SHELXS97* (Sheldrick, 2008 ▶); program(s) used to refine structure: *SHELXL97* (Sheldrick, 2008 ▶); molecular graphics: *ORTEP-3 for Windows* (Farrugia, 1997 ▶); software used to prepare material for publication: *WinGX* (Farrugia, 1999 ▶).

## Supplementary Material

Crystal structure: contains datablocks I, global. DOI: 10.1107/S1600536810017551/xu2761sup1.cif

Structure factors: contains datablocks I. DOI: 10.1107/S1600536810017551/xu2761Isup2.hkl

Additional supplementary materials:  crystallographic information; 3D view; checkCIF report

## Figures and Tables

**Table 1:** Selected bond lengths (Å)

Zn1—N1	2.157 (4)
Zn1—N2	2.141 (4)
Zn1—O1	2.125 (4)
Zn1—Br1	2.4701 (8)
Zn1—Br2	2.4148 (8)

**Table 2:** Hydrogen-bond geometry (Å, °)

*D*—H⋯*A*	*D*—H	H⋯*A*	*D*⋯*A*	*D*—H⋯*A*
C1—H1⋯Br1	0.93	2.86	3.413 (6)	119
C10—H10⋯O1	0.93	2.53	2.979 (7)	110

**Fig. 1.**
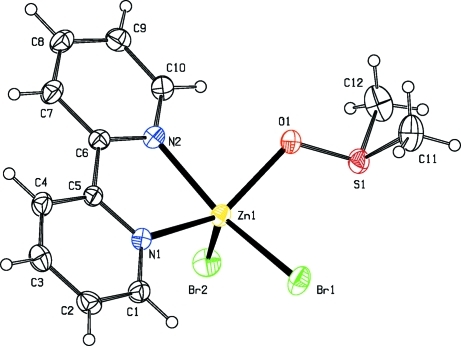
The molecular structure of the title molecule, with the atom-numbering scheme. Displacement ellipsoids are drawn at the 30% probability level.

**Fig. 2.**
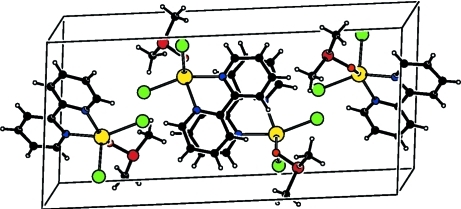
Unit-cell packing diagram for (I).

## supplementary crystallographic information

### Comment

There are several Zn^II^ complexes containing 2,2'-bipyridine and 2,2'-bipyridine derivatives such as, [ZnCl_2_(bipy)], (II), (Khan & Tuck, 1984), [ZnCl_2_(5,5'-dmbpy)], (III), (Khalighi *et al.*, 2008), [ZnCl_2_(6-mbpy)], (IV), (Ahmadi, *et al.*, 2008), [ZnCl_2_(6,6'-dmbpy)], (V), (Alizadeh, Kalateh, Ebadi, *et al.*, 2009), [ZnBr_2_(6,6'-dmbpy)], (VI), (Alizadeh, Khoshtarkib *et al.*, 2009), [ZnI_2_(6,6'-dmbpy)], (VII), (Alizadeh, Kalateh, Khoshtarkib *et al.*, 2009), [ZnCl_2_(bipy)(DMSO)], (VIII), (Marjani *et al.*, 2007) and [ZnBr_2_(4,4'-(dtbpy)].(Et_2_O), (IX), (Blake *et al.*, 2007) [where bipy is 2,2'-bipyridine, 5,5'-dmbpy is 5,5'-dimethyl-2,2'-bipyridine, 6,6'-dmbpy is 6,6'-dimethyl-2,2'-bipyridine, DMSO is dimethyl sulfoxide and dtbpy is 4,4'-di-tert-butyl-2,2'-bipyridine] have been synthesized and characterized by single-crystal X-ray diffraction methods. We report herein the synthesis and crystal structure of the title compound (I).

In the molecule of the title compound, (I), (Fig. 1), the Zn^II^ atom is five-coordinate in a distorted trigonal-bipyramidal configurations by two N atoms from one 2,2'-bipyridine, one atom from one dimethyl sulfoxide and two Br atoms. The Zn—N and Zn—O bond lengths and angles (Table 1) are within normal range (VIII) and Zn—Br bond lengths and angles are within normal range (VI).

The π-π contacts between the pyridine rings, Cg1···Cg3^i^, Cg2···Cg2^ii^, Cg2···Cg3^i^, Cg3···Cg1^i^ and Cg3···Cg2^i^ [symmetry cods: (i) 1-X,1-Y,1-Z, (ii) 1-X,-Y,1-Z, where Cg1, Cg2 and Cg3 are centroids of the rings (Zn1/N1/C5—C6/N2), (N1/C1—C5) and (N2/C6—C10), respectively] with centroid-centroid distance of 3.475 (3), 3.661 (3), 3.721 (3), 3.476 (3) and 3.721 (3) Å, respectively, and intramolecular C—H···O and C—H···Br hydrogen bonding it seems effective in the stabilization of the crystal structure (Fig. 2).

### Experimental

For the preparation of the title compound, (I), a solution of 2,2'-bipyridine (0.17 g, 1.10 mmol) in methanol (10 ml) was added to a solution of ZnBr_2_ (0.25 g, 1.10 mmol) in methanol (5 ml) at room temperature. The suitable crystals for X-ray diffraction experiment were obtained by methanol diffusion to a colorless solution in DMSO. Suitable crystals were isolated after one week (yield; 0.36 g, 71.2%).

### Refinement

H atoms were positioned geometrically with C—H = 0.93 Å for aromatic and 0.96 Å for methyl, and constrained to ride on their parent atoms with U_iso_(H)=1.2U_eq_(C).

### Crystal data

**Table:**

[ZnBr_2_(C_10_H_8_N_2_)(C_2_H_6_OS)]	*F*(000) = 896
*M**_r_* = 459.51	*D*_x_ = 1.938 Mg m^−^^3^
Monoclinic, *P*2_1_/*c*	Mo *K*α radiation, λ = 0.71073 Å
Hall symbol: -P 2ybc	Cell parameters from 653 reflections
*a* = 9.4802 (10) Å	θ = 2.1–29.2°
*b* = 8.3449 (7) Å	µ = 6.76 mm^−^^1^
*c* = 19.989 (2) Å	*T* = 298 K
β = 95.185 (8)°	Block, colorless
*V* = 1574.9 (3) Å^3^	0.30 × 0.25 × 0.20 mm
*Z* = 4	

### Data collection

**Table:**

Bruker SMART CCD area-detector diffractometer	4245 independent reflections
Radiation source: fine-focus sealed tube	3229 reflections with *I* > 2σ(*I*)
graphite	*R*_int_ = 0.078
φ and ω scans	θ_max_ = 29.2°, θ_min_ = 2.1°
Absorption correction: multi-scan (*SADABS*; Bruker, 2001)	*h* = −12→12
*T*_min_ = 0.148, *T*_max_ = 0.260	*k* = −11→11
12674 measured reflections	*l* = −27→25

### Refinement

**Table:**

Refinement on *F*^2^	Secondary atom site location: difference Fourier map
Least-squares matrix: full	Hydrogen site location: inferred from neighbouring sites
*R*[*F*^2^ > 2σ(*F*^2^)] = 0.058	H-atom parameters constrained
*wR*(*F*^2^) = 0.140	*w* = 1/[σ^2^(*F*_o_^2^) + (0.0602*P*)^2^ + 1.8278*P*] where *P* = (*F*_o_^2^ + 2*F*_c_^2^)/3
*S* = 1.13	(Δ/σ)_max_ = 0.005
4245 reflections	Δρ_max_ = 1.08 e Å^−^^3^
175 parameters	Δρ_min_ = −1.46 e Å^−^^3^
0 restraints	Extinction correction: *SHELXL97* (Sheldrick, 2008), Fc^*^=kFc[1+0.001xFc^2^λ^3^/sin(2θ)]^-1/4^
Primary atom site location: structure-invariant direct methods	Extinction coefficient: 0.0087 (7)

### Special details

**Table:**

Geometry. All esds (except the esd in the dihedral angle between two l.s. planes) are estimated using the full covariance matrix. The cell esds are taken into account individually in the estimation of esds in distances, angles and torsion angles; correlations between esds in cell parameters are only used when they are defined by crystal symmetry. An approximate (isotropic) treatment of cell esds is used for estimating esds involving l.s. planes.
Refinement. Refinement of *F*^2^ against ALL reflections. The weighted *R*-factor wR and goodness of fit *S* are based on *F*^2^, conventional *R*-factors *R* are based on *F*, with *F* set to zero for negative *F*^2^. The threshold expression of *F*^2^ > σ(*F*^2^) is used only for calculating *R*-factors(gt) etc. and is not relevant to the choice of reflections for refinement. *R*-factors based on *F*^2^ are statistically about twice as large as those based on *F*, and *R*- factors based on ALL data will be even larger.

### Fractional atomic coordinates and isotropic or equivalent isotropic displacement parameters (Å^2^)

**Table:**

	*x*	*y*	*z*	*U*_iso_*/*U*_eq_	
C1	0.5556 (5)	0.1151 (7)	0.3831 (3)	0.0377 (10)	
H1	0.5286	0.0980	0.3378	0.045*	
C2	0.6783 (5)	0.0424 (7)	0.4117 (3)	0.0425 (12)	
H2	0.7333	−0.0208	0.3859	0.051*	
C3	0.7166 (6)	0.0660 (7)	0.4788 (3)	0.0462 (13)	
H3	0.7982	0.0185	0.4992	0.055*	
C4	0.6335 (5)	0.1603 (6)	0.5155 (3)	0.0383 (10)	
H4	0.6581	0.1772	0.5611	0.046*	
C5	0.5129 (5)	0.2297 (6)	0.4840 (2)	0.0295 (9)	
C6	0.4154 (5)	0.3323 (6)	0.5190 (2)	0.0311 (9)	
C7	0.4311 (6)	0.3600 (7)	0.5883 (2)	0.0406 (11)	
H7	0.5056	0.3137	0.6150	0.049*	
C8	0.3346 (6)	0.4570 (7)	0.6166 (3)	0.0432 (12)	
H8	0.3433	0.4771	0.6625	0.052*	
C9	0.2254 (5)	0.5234 (7)	0.5759 (3)	0.0422 (12)	
H9	0.1589	0.5889	0.5939	0.051*	
C10	0.2162 (5)	0.4915 (7)	0.5085 (3)	0.0395 (11)	
H10	0.1421	0.5366	0.4812	0.047*	
C11	0.2234 (7)	0.7783 (10)	0.2635 (4)	0.0621 (18)	
H11A	0.3167	0.7412	0.2564	0.075*	
H11B	0.2302	0.8683	0.2935	0.075*	
H11C	0.1750	0.8099	0.2213	0.075*	
C12	−0.0109 (6)	0.7414 (10)	0.3256 (4)	0.0650 (19)	
H12A	0.0281	0.8281	0.3529	0.078*	
H12B	−0.0698	0.6765	0.3514	0.078*	
H12C	−0.0665	0.7838	0.2872	0.078*	
N1	0.4750 (4)	0.2084 (5)	0.41813 (19)	0.0324 (8)	
N2	0.3090 (4)	0.3984 (5)	0.48005 (19)	0.0324 (8)	
O1	0.2163 (4)	0.5774 (5)	0.36384 (18)	0.0440 (9)	
Zn1	0.29167 (5)	0.33824 (7)	0.37552 (3)	0.03244 (16)	
Br1	0.37506 (6)	0.34269 (8)	0.26199 (3)	0.04918 (19)	
Br2	0.07887 (6)	0.17861 (8)	0.37356 (3)	0.05171 (19)	
S1	0.12833 (14)	0.62307 (17)	0.29876 (7)	0.0413 (3)	

### Atomic displacement parameters (Å^2^)

**Table:**

	*U*^11^	*U*^22^	*U*^33^	*U*^12^	*U*^13^	*U*^23^
C1	0.040 (2)	0.040 (3)	0.033 (2)	0.006 (2)	0.0043 (19)	−0.001 (2)
C2	0.042 (3)	0.038 (3)	0.049 (3)	0.009 (2)	0.012 (2)	−0.001 (2)
C3	0.041 (3)	0.044 (3)	0.052 (3)	0.009 (2)	−0.002 (2)	0.008 (3)
C4	0.038 (2)	0.041 (3)	0.035 (2)	−0.001 (2)	−0.0051 (18)	0.004 (2)
C5	0.032 (2)	0.028 (2)	0.028 (2)	−0.0061 (17)	0.0007 (16)	0.0022 (17)
C6	0.0297 (19)	0.031 (2)	0.032 (2)	−0.0062 (17)	0.0025 (16)	−0.0013 (19)
C7	0.043 (3)	0.047 (3)	0.031 (2)	−0.006 (2)	−0.0026 (19)	−0.001 (2)
C8	0.052 (3)	0.049 (3)	0.029 (2)	−0.008 (2)	0.008 (2)	−0.003 (2)
C9	0.046 (3)	0.040 (3)	0.043 (3)	0.000 (2)	0.018 (2)	−0.001 (2)
C10	0.037 (2)	0.043 (3)	0.039 (2)	0.005 (2)	0.0079 (19)	0.004 (2)
C11	0.047 (3)	0.080 (5)	0.060 (4)	−0.005 (3)	0.010 (3)	0.026 (4)
C12	0.040 (3)	0.071 (5)	0.086 (5)	0.006 (3)	0.014 (3)	0.022 (4)
N1	0.0333 (18)	0.034 (2)	0.0299 (18)	0.0003 (15)	0.0015 (14)	0.0026 (16)
N2	0.0278 (17)	0.039 (2)	0.0309 (18)	−0.0022 (15)	0.0029 (14)	−0.0005 (17)
O1	0.053 (2)	0.041 (2)	0.0352 (18)	0.0093 (16)	−0.0089 (15)	0.0020 (16)
Zn1	0.0303 (3)	0.0378 (3)	0.0285 (3)	0.0010 (2)	−0.00125 (19)	0.0005 (2)
Br1	0.0467 (3)	0.0704 (4)	0.0309 (2)	0.0102 (3)	0.0059 (2)	0.0055 (3)
Br2	0.0373 (3)	0.0562 (4)	0.0604 (4)	−0.0122 (2)	−0.0025 (2)	−0.0009 (3)
S1	0.0409 (6)	0.0409 (7)	0.0397 (6)	0.0015 (5)	−0.0084 (5)	0.0036 (5)

### Geometric parameters (Å, °)

**Table:**

C1—N1	1.333 (6)	C9—H9	0.9300
C1—C2	1.388 (7)	C10—N2	1.338 (6)
C1—H1	0.9300	C10—H10	0.9300
C2—C3	1.372 (8)	C11—S1	1.761 (7)
C2—H2	0.9300	C11—H11A	0.9600
C3—C4	1.372 (8)	C11—H11B	0.9600
C3—H3	0.9300	C11—H11C	0.9600
C4—C5	1.382 (6)	C12—S1	1.770 (7)
C4—H4	0.9300	C12—H12A	0.9600
C5—N1	1.345 (6)	C12—H12B	0.9600
C5—C6	1.480 (6)	C12—H12C	0.9600
C6—N2	1.336 (6)	Zn1—N1	2.157 (4)
C6—C7	1.401 (6)	Zn1—N2	2.141 (4)
C7—C8	1.380 (8)	S1—O1	1.529 (4)
C7—H7	0.9300	Zn1—O1	2.125 (4)
C8—C9	1.374 (8)	Zn1—Br1	2.4701 (8)
C8—H8	0.9300	Zn1—Br2	2.4148 (8)
C9—C10	1.369 (7)		
			
N1—C1—C2	122.6 (5)	S1—C11—H11B	109.5
N1—C1—H1	118.7	H11A—C11—H11B	109.5
C2—C1—H1	118.7	S1—C11—H11C	109.5
C3—C2—C1	118.5 (5)	H11A—C11—H11C	109.5
C3—C2—H2	120.7	H11B—C11—H11C	109.5
C1—C2—H2	120.7	S1—C12—H12A	109.5
C2—C3—C4	119.4 (5)	S1—C12—H12B	109.5
C2—C3—H3	120.3	H12A—C12—H12B	109.5
C4—C3—H3	120.3	S1—C12—H12C	109.5
C3—C4—C5	119.2 (5)	H12A—C12—H12C	109.5
C3—C4—H4	120.4	H12B—C12—H12C	109.5
C5—C4—H4	120.4	C1—N1—C5	118.4 (4)
N1—C5—C4	121.8 (5)	C1—N1—Zn1	124.5 (3)
N1—C5—C6	114.5 (4)	C5—N1—Zn1	117.1 (3)
C4—C5—C6	123.6 (4)	C6—N2—C10	118.9 (4)
N2—C6—C7	121.0 (4)	C6—N2—Zn1	117.3 (3)
N2—C6—C5	115.8 (4)	C10—N2—Zn1	123.7 (3)
C7—C6—C5	123.2 (4)	S1—O1—Zn1	118.7 (2)
C8—C7—C6	119.1 (5)	O1—Zn1—N2	83.24 (15)
C8—C7—H7	120.4	O1—Zn1—N1	139.98 (15)
C6—C7—H7	120.4	N2—Zn1—N1	75.19 (15)
C9—C8—C7	119.1 (5)	O1—Zn1—Br2	104.13 (11)
C9—C8—H8	120.4	N2—Zn1—Br2	97.80 (11)
C7—C8—H8	120.4	N1—Zn1—Br2	111.81 (11)
C10—C9—C8	118.8 (5)	O1—Zn1—Br1	90.99 (11)
C10—C9—H9	120.6	N2—Zn1—Br1	152.93 (11)
C8—C9—H9	120.6	N1—Zn1—Br1	93.27 (11)
N2—C10—C9	123.1 (5)	Br2—Zn1—Br1	109.25 (3)
N2—C10—H10	118.5	O1—S1—C11	105.3 (3)
C9—C10—H10	118.5	O1—S1—C12	104.2 (3)
S1—C11—H11A	109.5	C11—S1—C12	97.7 (4)
			
N1—C1—C2—C3	1.0 (9)	C9—C10—N2—C6	0.4 (8)
C1—C2—C3—C4	−0.2 (9)	C9—C10—N2—Zn1	177.1 (4)
C2—C3—C4—C5	−0.1 (8)	S1—O1—Zn1—N2	−159.5 (3)
C3—C4—C5—N1	−0.5 (8)	S1—O1—Zn1—N1	143.3 (2)
C3—C4—C5—C6	179.5 (5)	S1—O1—Zn1—Br2	−63.2 (3)
N1—C5—C6—N2	−4.3 (6)	S1—O1—Zn1—Br1	47.0 (3)
C4—C5—C6—N2	175.7 (4)	C6—N2—Zn1—O1	−146.3 (4)
N1—C5—C6—C7	175.5 (4)	C10—N2—Zn1—O1	36.9 (4)
C4—C5—C6—C7	−4.5 (7)	C6—N2—Zn1—N1	−0.3 (3)
N2—C6—C7—C8	0.1 (8)	C10—N2—Zn1—N1	−177.1 (4)
C5—C6—C7—C8	−179.7 (5)	C6—N2—Zn1—Br2	110.3 (3)
C6—C7—C8—C9	0.2 (8)	C10—N2—Zn1—Br2	−66.5 (4)
C7—C8—C9—C10	−0.2 (8)	C6—N2—Zn1—Br1	−67.5 (5)
C8—C9—C10—N2	−0.1 (8)	C10—N2—Zn1—Br1	115.7 (4)
C2—C1—N1—C5	−1.5 (8)	C1—N1—Zn1—O1	−119.4 (4)
C2—C1—N1—Zn1	175.5 (4)	C5—N1—Zn1—O1	57.6 (4)
C4—C5—N1—C1	1.2 (7)	C1—N1—Zn1—N2	−179.1 (4)
C6—C5—N1—C1	−178.8 (4)	C5—N1—Zn1—N2	−2.2 (3)
C4—C5—N1—Zn1	−175.9 (4)	C1—N1—Zn1—Br2	88.3 (4)
C6—C5—N1—Zn1	4.0 (5)	C5—N1—Zn1—Br2	−94.7 (3)
C7—C6—N2—C10	−0.4 (7)	C1—N1—Zn1—Br1	−24.0 (4)
C5—C6—N2—C10	179.4 (4)	C5—N1—Zn1—Br1	153.0 (3)
C7—C6—N2—Zn1	−177.3 (4)	Zn1—O1—S1—C11	−120.4 (4)
C5—C6—N2—Zn1	2.5 (5)	Zn1—O1—S1—C12	137.3 (3)

### Hydrogen-bond geometry (Å, °)

**Table:**

*D*—H···*A*	*D*—H	H···*A*	*D*···*A*	*D*—H···*A*
C1—H1···Br1	0.93	2.86	3.413 (6)	119
C10—H10···O1	0.93	2.53	2.979 (7)	110
